# Pressure-induced high-spin/low-spin disproportionated state in the Mott insulator FeBO_3_

**DOI:** 10.1038/s41598-022-13507-4

**Published:** 2022-06-10

**Authors:** Weiming Xu, Weiwei Dong, Samar Layek, Mark Shulman, Konstantin Glazyrin, Elena Bykova, Maxim Bykov, Michael Hanfland, Moshe P. Pasternak, Ivan Leonov, Eran Greenberg, Gregory Kh. Rozenberg

**Affiliations:** 1 School of Physics and Astronomy, Tel-Aviv University, 69978 Tel-Aviv, Israel; 2 Deutsches Elektronen Synchrotron (DESY), Notkestr. 85, 22607 Hamburg, Germany; 3 Department of Physics, School of Engineering, University of Petroleum and Energy Studies (UPES), Dehradun, Uttarakhand 248007 India; 4 Earth and Planets Laboratory, Carnegie Institution for Science, Washington, DC 20015 USA; 5 European Synchrotron Radiation Facility, BP220, 38043 Grenoble, France; 6 M.N. Miheev Institute of Metal Physics, Russian Academy of Sciences, 620108 Yekaterinburg, Russia; 7 Ural Federal University, 620002 Yekaterinburg, Russia; 8 Applied Physics Division, Soreq NRC, 81800 Yavne, Israel

**Keywords:** Materials science, Physics

## Abstract

The pressure-induced Mott insulator-to-metal transitions are often accompanied by a collapse of magnetic interactions associated with delocalization of 3*d* electrons and high-spin to low-spin (HS-LS) state transition. Here, we address a long-standing controversy regarding the high-pressure behavior of an archetypal Mott insulator FeBO_3_ and show the insufficiency of a standard theoretical approach assuming a conventional HS-LS transition for the description of the electronic properties of the Mott insulators at high pressures. Using high-resolution x-ray diffraction measurements supplemented by Mössbauer spectroscopy up to pressures ~ 150 GPa, we document an unusual electronic state characterized by a “mixed” HS/LS state with a stable abundance ratio realized in the $R\overline{3 }c$ crystal structure with a single Fe site within a wide pressure range of ~ 50–106 GPa. Our results imply an unconventional cooperative (and probably dynamical) nature of the ordering of the HS/LS Fe sites randomly distributed over the lattice, resulting in frustration of magnetic moments.

## Introduction

Pressure-induced electronic and magnetic phase transitions in 3*d* transition metal compounds have been a widespread research topic over the past decades, being especially relevant to the understanding of high-temperature superconductivity, metal–insulator transitions, colossal magnetoresistance, and heavy-fermion behavior^1–4^. At ambient pressure many of these compounds belong to the broad class of Mott insulators^5^, whose behavior is a result of strong on-site Coulomb repulsion between the *3d* electrons that is not mitigated by the restricted range of kinetic energies available for the narrow 3*d-*band system. One of the most fascinating electronic transformations in such compounds is the breakdown of the 3*d* electron localization resulting in a Mott insulator-to-metal phase transition (IMT) which is usually accompanied by a collapse of magnetic interactions^1,2^. The Mott IMT has been the subject of numerous high-pressure studies, particularly of iron-bearing oxides (^6^ and ref. therein), using conventional and synchrotron-based Mössbauer spectroscopy (MS) in combination with diamond anvil cell (DACs) techniques^7^. The initial concept of Mott is based on a relative importance of kinetic hopping and on-site Coulomb repulsion of the 3*d* electrons. However, in addition, it has been proposed that a change of the crystal-field splitting, or a decrease of the effective interaction strength caused by a high-to-low spin (HS-LS) crossover can drive a Mott–Hubbard transition^8–12^. As a result, the Mott transition is accompanied by a concurrent insulator-to-metal and local spin state transition, resulting in a collapse of the lattice volume. In ferric compounds, a typical pressure range of the HS-LS crossover for Fe^3+^ ion in an octahedral environment ~ 40–60 GPa^6,11–13^ and consequently above this pressure the material is usually a metal or a narrow-gap semiconductor, turning to a metal upon further compression^9–12^. Nevertheless, recent theoretical calculations imply that in many cases more complex scenarios can emerge, different from the generally accepted models of a HS-LS transition, suggesting a crucial importance of correlation effects in understanding the electronic/magnetic transformations under pressure^14–16^.

Iron borate, FeBO_3_, is one of a few materials that are transparent in a broad range of visible light and have a spontaneous magnetization at room temperature, which makes it attractive in applications for visible or x-ray light magneto-optical devices^17^. It is a canted antiferromagnet with the Néel temperature *T*_N_ ~ 348 K and weak ferromagnetism^18^. Optical spectroscopy shows that FeBO_3_ is a Mott insulator with a large energy gap of ~ 2.9 eV (^19^ and ref. therein). Formally, FeBO_3_ can be considered as a part of a more general Fe*X*O_3_ family (e.g., FeFeO_3_, FeGaO_3_, etc.), with ferric Fe^3+^ ion playing a major role in determining the electronic and magnetic properties of Fe*X*O_3_. Recent extensive high-pressure studies of FeBO_3_ reveal that, similar to many other ferrites, in the vicinity of ~ 50 GPa it undergoes an isostructural phase transition corroborating with a significant reduction of the lattice volume and coinciding with an abrupt magnetic collapse and a Mott insulator-to-semiconductor transition^19–22^. However, we notice that FeBO_3_ shows some very specific features following an affirmed HS-LS transition at ~ 50 GPa^21,22^. Most notably, it exhibits apparently resilient non-metallic behavior above 100 GPa^19^. This behavior is different, e.g., to FeGaO_3_ and Fe_2_O_3_ which exhibit the classical band-width controlled Mott transition at ~ 50 GPa, characterized by a complete collapse of magnetic interactions^23^, and a site-selective Mott IMT at a similar pressure range^24,25^, respectively. In this regard, FeBO_3_ and its high-pressure behavior is of particular interest as a possible example material documenting different mechanisms for electronic transitions.

In our work, we present a detailed study of the electronic structure, local magnetic state of Fe^3+^ ions, and phase stability of FeBO_3_ up to pressures ~ 150 GPa, combining room and low-temperature ^57^Fe Mössbauer spectroscopy with single crystal (SC) and powder (PWD) X-ray diffraction. Our results reveal that the simultaneous magnetic and isostructural transition in FeBO_3_ at ~ 50 GPa, which was previously considered as a HS-LS transition^20,21^, is in fact the transition to a “mixed” HS/LS state. It is characterized by a stable (although a weakly temperature-dependent) abundance ratio of the HS/LS states of ~ 1:3 in a wide pressure range of ~ 50–106 GPa. Our observations are unexpected given the presence of only a single Fe^3+^ structural position in the crystal structure of FeBO_3_. This behavior clearly distinguishes the behavior in FeBO_3_ from a “conventional” spin-state crossover observed in other ferric systems. We propose a model explaining this unusual electronic state based on a complex interplay between the spin and lattice degrees of freedom.

## Results

### X-ray diffraction

**Figure 1:**
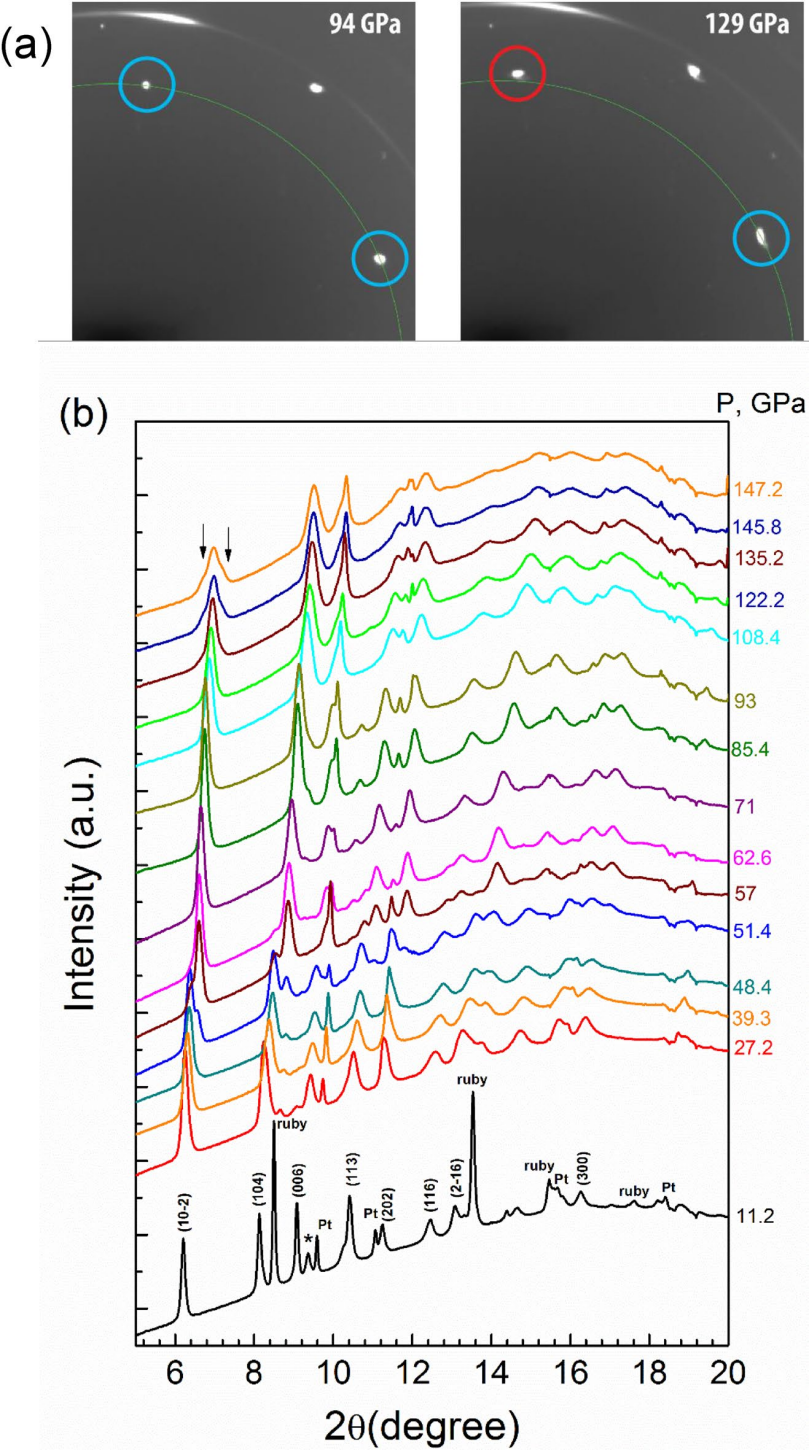
X-ray single crystal (**a**) and powder (**b**) diffraction patterns of FeBO_3_ at RT at various pressures (λ = 0.2898 Å and 0.3738 Å, respectively). Note a splitting of the $(10\overline{2 })$ reflection in the SC and powder XRD pattern at 129 and 145.8 GPa, respectively, signifying lowering of the original symmetry. * marks an unidentified peak, which disappears at higher pressures.

**Figure 2:**
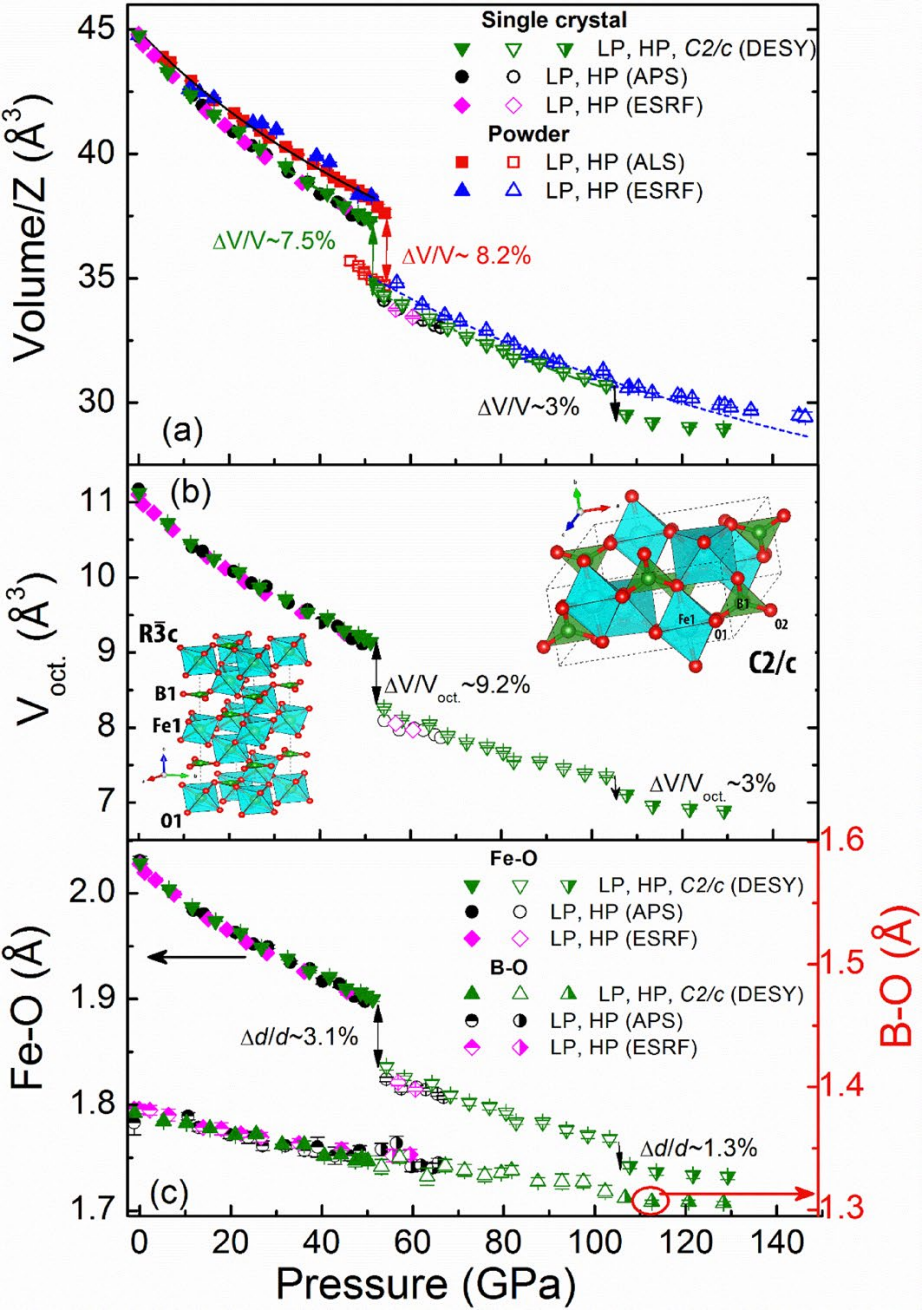
Pressure dependencies of the unit-cell volume divided by *Z* unit formulas (Z = 6 and 4 for the $R\overline{3}\mathrm{c }$ and *C2/c* phases, respectively) (**a**), determined in the powder and single crystal XRD studies; FeO_6_ octahedral volume (**b**) and average Fe–O, B-O distances (**c**) for FeBO_3_. The solid, dashed, dash-dot and short-dash lines in (**a**) are fits with the Birch-Murnaghan equation of state (see text). The panel (**b**) insets show the $R\overline{3}\mathrm{c }$ and *C2/c* crystal structures, respectively. The red and green spheres correspond to the oxygen and boron atoms.

We perform four independent SC-XRD experiments, which are mutually consistent for their measured pressure range. The exceptional quality of the data is reflected in the parameters of fit (see Supplementary information). In particular, we found that at ambient pressure FeBO_3_ adopts the rhombohedral $R\overline{3}\mathrm{c }$ crystal structure (see inset Fig. 2b and Supplementary Fig. S2), in agreement with previous PWD and SC XRD [20 and ref. therein]. The $R\overline{3}\mathrm{c }$ structure is conserved at least up to ~ 105 GPa (Figs. 1, 2). At about 50 GPa we observe a doubling of the reflections within the diffraction patterns attributed to the onset of the high-pressure (HP) phase characterized by the same space group ($R\overline{3}\mathrm{c }$) but with significantly reduced unit-cell volume and lattice parameters (Supplementary Fig. S1). Our SC-XRD data confirm that multiple domains of the HP phase grow on top of the HS low-pressure (LP) state grains preserving the same orientation. Here we extend the previous PWD-XRD studies^20^ and shed new light on the process of phase transformations in FeBO_3_. We observe a finite range (~ 50–55 GPa) of the HP-LP phase coexistence on the same grains. Although, this isostructural phase transition is related to an increased strain and broadening of the peaks with a slight increase of mosaicity, as well as of dislocation density, we were able to solve the crystal structure as a function of pressure, extracting structural parameters with a high precision.

Our results for the compressional behavior of the LP $R\overline{3 }c$ phase *V*(*P*) are fitted with a second-order Birch-Murnaghan (BM2) equation of state (EOS)^26^ (see Fig. 2a). At 1 bar and 298 K the calculated bulk modulus and equilibrium unit-cell volume are *K*_0_ = 200.14(7) GPa and *V*_0_ = 268.2(2) Å^3^, respectively. For the HP $R\overline{3 }c$ phase we obtain *K*_0_ = 153.8(7) GPa and *V*_0_ = 257.5(8) Å^3^ The volume and bulk modulus at 50 GPa (*V*_50_ and *K*_50_) for the LP phase are 224.98 Å^3^ and 384.9 GPa and 230.33 Å^3^ and 417.2 GPa for SC and PWD, respectively. For the HP phase the values are 208.22 Å^3^ and 335.8 GPa and 211.67 Å^3^ and 343.5 GPa for SC and PWD, respectively. We observe a large unit-cell volume reduction at the isostructural transition above ~ 50 GPa, Δ*V/V* ~ 7.5%. It is attributed to the shrinkage of the Fe–O interatomic distances and the corresponding decrease of the FeO_6_ octahedral volume (Fig. 2b,c).

At about 106 GPa we observe a splitting of the $(10\overline{2 })$ reflection (in hexagonal notation) in the SC-XRD pattern (see Fig. 1a,b, Supplementary Fig. S3), implying a structural phase transition with a lowering of the unit-cell symmetry. Based on the SC-XRD we solve the new structure (designated as HP2) to be the monoclinic with the space group *C2/c* (see inset Fig. 2b), a subgroup of the original $R\overline{3}\mathrm{c }$, with a single Fe site (see Supplementary Fig. S6 in which we show relations between the $R\overline{3}\mathrm{c }$ and *C2/c* unit-cells in the instrumental cartesian coordinates). The phase transition is accompanied by a lattice volume contraction of ~ 3%.

Similar to $R\overline{3}\mathrm{c }$, the *C2/c* phase consists of distorted FeO_6_ octahedrons and BO_3_ triangles. The B-O distances are found to decrease almost linearly as a function of pressure. We notice a small increase of the B-O distances by ~ 0.5% at the transition to the HP phase. Upon the transition to the *C2/c* structure the B-O distances decrease at a much slower pace than before. Thus, considering the data shown in Fig. 2c, the major transformation is associated with ferric cations.

In comparison to SC-XRD, for the PWD data (Fig. 2a) we observe a larger unit-cell volume at a given pressure, larger bulk modulus values for the LP phase, and a broader pressure range for the phase transition around 50 GPa; all these are indicative of deviatoric stress and enhanced strain contributions, i.e., grain-grain interactions (for more details see Supplementary and Refs.^27–32^). However, comparing the SC and PWD data we notice overall similarity of the *V(P)* behavior at pressures below ~ 100 GPa. At higher pressures, splitting of the $(10\overline{2 })$ reflection appears at ~ 130 GPa, slowly developing and eventually becoming clearly distinguishable around 145 GPa. However, due to the peak broadness and incompleteness of the phase transition, the phases could not be reliably separated even at ~ 145 GPa. It is noteworthy, that an appreciable deviation of PWD *V(P)* data from the EOS, calculated for the HP $R\overline{3}\mathrm{c }$ phase below 115 GPa, is observed from ~ 110 GPa (Fig. 2a, Supplementary Fig. S7). It is not very different from the pressure of the $R\overline{3 }c$→*C2/c* phase transition in the SC-XRD. (The pressure range was limited to ~ 115 GPa since its extension to higher pressures leads to degradation of the EOS fit quality and results in a significant deviation of the obtained parameters from those calculated for SC EOS. For 60–115 GPa range the performed fit using a BM2 EOS results in *K*_0_ = 149(12) GPa, *V*_0_ = 262(2) Å^3^ suggesting an appreciable “pressure overestimation” in PWD measurements (see^27–30^).)

### Mössbauer spectroscopy

**Figure 3:**
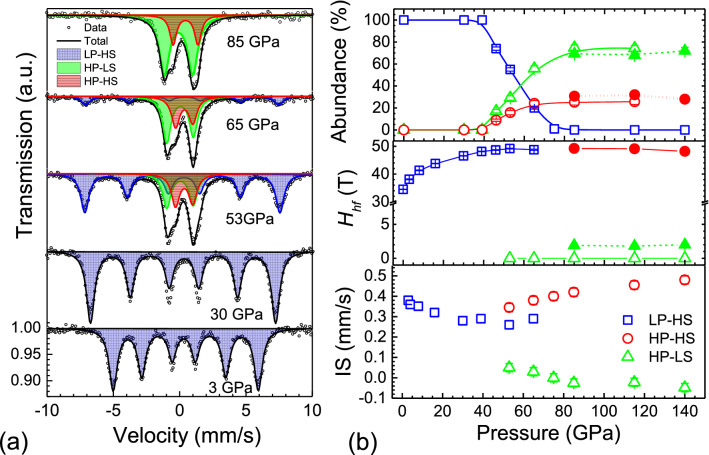
(**a**) Mössbauer spectra of FeBO_3_ at various pressures and room temperature. Empty circles represent experimental data points whereas the black solid line through the data points represents the overall fit to the data from the sum of sub-components shown. The blue and orange shaded sub-components refer to LP-HS-and HP-HS states, whereas the green one refers to HP-LS. (**b**) Pressure-dependence of the isomer shift (IS), hyperfine field (*H*_hf_) and abundances (or area percentage) extracted from best fits to the Mössbauer spectra. Solid symbols indicate the values extracted from low-temperature measurements (3–10 K); IS values at 140 GPa correspond to *T* = 150 K.

In Fig. 3 we display Mössbauer spectra of polycrystalline FeBO_3_ for different pressures recorded at room temperature (RT). In agreement with previous publications^22^, the only observed spectral component upon compression up to ~ 45 GPa within the LP phase is that of the HS state (LP-HS, *S* = 5/2, ^6^*A*_1*g*_) characterized by a magnetically-split sextet and a small quadrupole splitting (QS ≈ 0 mm/s). At *P* ≥ 46 GPa two new doublets emerge: (1) a more intense component with QS ≈ 2 mm/s and small isomer shift (IS) ≈ 0.03 mm/s; and (2) a less intense component with QS ≈ 1.7 mm/s and IS ≈ 0.34 mm/s. Correspondingly, within the coexistence range (~ 46–65 GPa) the spectra are the superposition of three components: the magnetically ordered LP phase with *H*_hf_ ~ 48 T and IS ≈ 0.27 mm/s, and two high-pressure doublets. We note that a similar observation was previously reported by Sarkisyan et al*.*^22^ for PWD FeBO_3_ at a limited pressure range of ~ 48–58 GPa Considering the Ref.^22^, the authors suggested different behavior of powder and single crystal data. According to their MS results, a two-doublet-structure is not evident in the SC sample up to ~ 55 GPa, in contrast to the powder. We note the rather poor statistics of the SC data at high pressures in Ref.^22^, which potentially prevented detecting small features. Indeed, there is a clear evidence of a small shoulder at 46.6 GPa in the vicinity of 0 mm/s in their Fig. 1 corresponding to single crystal measurements. In order to resolve a potential controversy, we performed our own experiment with large grain material and Ne pressure medium. In this experiment, we tried to avoid possible preferred orientation effects, and, instead of one big piece of a single crystal^22^, we measured tens of pieces of about 10 μm size, obtained by crushing of a SC sample. Noteworthy, in this experiment we did not detect any appreciable difference compared to the case of a powder sample grinded to a size of ~ 1 μm. This MS result in conjunction with the XRD results suggests that the differences between the powder and SC cases are rather small to claim as different scenarios in these cases.

Upon compression above ~ 65 GPa the magnetically-split LP component disappears and only quadrupole-split components (1) and (2) are observed at RT (Fig. 3) with almost the same abundances ratio of ~ 3:1. The parameters QS and IS of the more intense doublet are typical for that of the LS Fe^3+^ state (*S* = 1/2, ^6^*T*_2*g*_) and coincide with the values obtained for the high-pressure Fe^3+^ state in SC FeBO_3_^22^. At the same time, the parameters of the less intense doublet are more typical for that in the Fe^3+^ HS state^33^. The pressure-dependencies of the isomer shift of the various components and their relative abundances are summarized in Fig. 3b The relative abundance of site *i* was determined from the respective areas *A*_*i*_ under the absorption peaks for each component using the relation *A*_*i*_ = *Kn*_*i*_*f*_*i*_ where *K* is a constant, *n*_*i*_ is the abundance of component *i*, and *f*_*i*_ is its recoil-free fraction. We assumed as a first approximation that at each pressure the recoil-free fraction values *f*_*i*_ for the three components are the same.

**Figure 4:**
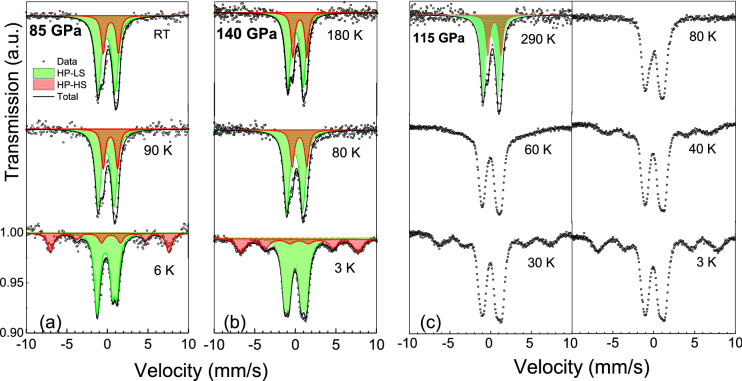
Mössbauer spectra of FeBO_3_ at different temperatures at 85 GPa (**a**), 140 GPa (**b**) and 115 GPa (**c**). Spectra at 115 and 140 GPa were collected using synchrotron MS. Spectra collected at 115 GPa allow us to define the Néel temperature of ~ 60(10) K.

To further clarify the nature of the HP MS components we perform low-temperature MS measurements at temperatures down to 3 K. In Fig. 4 we show the MS spectra for ~ 85 and 140 GPa collected at various temperatures using in-house and synchrotron Mössbauer spectroscopy. Our results reveal a magnetic splitting for both sites at low temperatures although characterized by a very different magnetic splitting: 0.57(5) and 14.75(5) mm/s (at 85 GPa); the corresponding hyperfine field values are *H*_hf_ = 1.9(2) and 49.5(2) T, respectively. Based on the obtained IS and *H*_hf_ values we finally attribute these components to the HP LS and HP HS states, respectively. We note that the relative abundances of these components slightly change upon cooling. In fact, the relative abundances of the HS state seem to slightly increase upon temperature decrease (see Fig. 3b). Our detailed low-temperature measurements performed at ~ 115 GPa (Fig. 4c) allow us to estimate the Néel temperature of the HP HS state. The obtained value of *T*_N_ ~ 60(10)K is significantly lower than the *T*_N_ ~ 600 K of LP HS state at ~ 50 GPa^21^. We note that for the HP phase *T*_N_ is almost the same for both components, HS and LS, and that our estimate for *T*_N_ is in good quantitative agreement with that obtained from the NFS data for the HP phase (~ 50 K at the range 50–55 GPa)^21^, suggesting that the antiferromagnetic state with *T*_N_ ~ 60 K persists in a broad pressure range of ~ 50–106 GPa. Our results therefore suggest that around 50 GPa the major portion of the Fe^3+^ ions in FeBO_3_ undergoes a transition into the LS state, while the rest of the Fe^3+^ ions remain in the HS state (paramagnetic at room temperature).

Interestingly, the phase transition in the mixed HS-LS state above ~ 50 GPa does not lead to a change of the unit-cell symmetry or superstructure formation^34,35^ in FeBO_3_. In fact, our high-resolution SC and PWD XRD with high accuracy show the *R*3̅c unit-cell symmetry characterized by a single Fe^3+^ site up to ~ 106 GPa, implying a cooperative dynamical ordering of the HS/LS Fe^3+^ sites.

## Discussion

Our data indicate that under ambient conditions and up to high compression ~ 106 GPa, the crystal lattice of FeBO_3_ has the rhombohedral symmetry with space group $R\overline{3}\mathrm{c }$ with a single crystallographic position of the Fe^3+^ ions. In agreement with previous studies^20^, at ~ 50 GPa FeBO_3_ undergoes an isostructural phase transition corroborating with a lattice volume collapse of ~ 7.5% We note differences in the onset pressures and the pressure range of spin crossover as discerned by XRD and MS, which may be attributed to the different pressure transmitting media used and how the degree of nonhydrostaticity affects the electronic transition (see Refs.^31,32^). In addition, we note that in the synchrotron XRD measurements the signal derives from a small central part of the sample, whereas in Mössbauer pressure studies the signal is collected from a much larger ∼2*/*3 inner region of the sample diameter. In the latter case this results in a potential importance of pressure gradient effects which, on top of the deviatoric stress effect, could be impactful in determining phase transition pressures and the transition pressure range. It was previously shown that at the transition the charge gap drops from ~ 2.9 to 0.5 eV and then gradually decreases within the HP phase^19^. Previously this transition was considered as a conventional (complete) HS–LS transition of all Fe^3+^ ions in FeBO_3_^21,22^. However, our high-resolution single crystal study gives previously inaccessible structural information which shows the inconsistency of this assumption. We report that the Fe–O distances are shortened by ~ 3.1% and the FeO_6_ octahedral volume drops by Δ*V/V*_oct_ ~ 9.2%. The octahedral volume reduction is remarkably smaller (by ~ 3%) compared to what is observed in confirmed complete Fe^3+^ HS–LS transitions, e.g., in CaFe_2_O_4_^36^ and FeOOH^37^, where Δ*V/V*_oct_ ~ 12–12.4% In Fe2O3 the octahedral volume change is even larger, of ~ 14%. However, in this case spin transition coincides with a structure change^38^. In contrast to FeBO_3_, the latter values are in good agreement with the theoretical values tabulated by Shannon^36,39^.

Our ambient temperature MS shows that despite the appearance of the LS state at ~ 50 GPa, a significant part of the Fe^3+^ ions remains in the HS state up to the highest pressures studied here. Most notably, above ~ 65 GPa the abundance of the HS state is almost unaffected by compression and remains rather high, even at pressures above ~ 100 GPa (see Fig. 4). The obtained MS results correlate with a change of the Fe^3+^ site volume. The ~ 3% deficiency in the octahedral volume change for the ~ 50 GPa transition is consistent with the idea of a *partial* spin transition. Our SC XRD and MS data reveal that for a wide range of pressures and at RT only 75(3)% of Fe^3+^ ions are in the LS state, while the rest remain in the HS state, i.e., the abundance ratio of the HS-to-LS states is of ~ 1:3.

**Figure 5:**
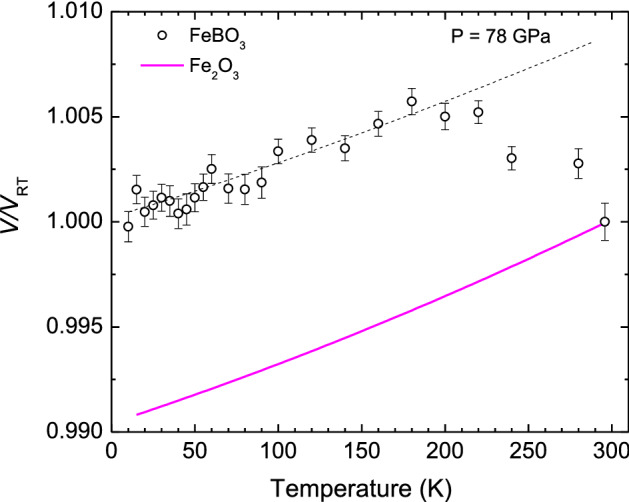
Temperature dependence of the relative unit-cell volume for FeBO_3_ at ~ 78 GPa. For comparison we show also the temperature dependence of the relative unit-cell volume for HS Fe_2_O_3_, which belong to the same Fe*X*O_3_ family, at ambient pressure (solid line) calculated from Ref.^40^. The dashed line is to guide the eye for a conventional thermal expansion behavior of FeBO_3_ at the range 10–180 K.

Furthermore, based on the low-temperature MS we verify that the HS/LS abundance ratio shows a weak temperature dependence. To further clarify this point we performed isobaric PWD-XRD measurements, testing the assumption that a change in this ratio may correlate with the unit-cell volume changes. In fact, our low-temperature measurements conducted at 78 GPa show an appreciable negative thermal expansion of the $R\overline{3}\mathrm{c }$ unit-cell at ~ 180–295 K (see Fig. 5, Supplementary Fig. S8) confirming a possible change of the HS/LS abundances ratio with temperature. Below ~ 180 K a conventional *V*(*T*) behavior is observed indicating stabilization of the HS/LS ratio. The observed negative thermal expansion is associated with a crystal volume change by ~ 0.9% upon cooling (Fig. 5), which suggests a rise by ~ 8% of the abundance of the HS state, in agreement with the MS data.

Our results therefore suggest a formation of a stable “mixed” spin state within a very broad pressure range of ~ 50–106 GPa in FeBO_3_. Realization of such steady spin-disproportionated state assumes a cooperative ordering of the Fe^3+^ sites with the HS and LS moments. The unforeseen coincidence of the Néel temperatures for the HS and LS states supports a cooperative nature of the mixed spin state. Moreover, since in the *R*3̅c structure all the Fe^3+^ occupy identical crystallographic sites, the site selection for HS and LS is random, and not fixed, which results in frustration of magnetic moments. This cooperative phenomenon could potentially be dynamic with dynamical correlations playing a major role within a wide pressure range preceding delocalization of the *3d* electrons. While it seems to be plausible that such a dynamical effect may arise from the instantaneous HS-LS interaction of electronic origin, this topic needs further detailed theoretical and experimental considerations^41,42^. Our numerous attempts to detect a possible formation of a superlattice (reduction of the unit-cell symmetry) in FeBO_3_ at ~ 50–106 GPa using SC-XRD have not been successful, which is consistent with the proposed dynamical features (critical nature) of spin correlations. We note that recent theoretical model calculations propose the formation of a (static) spin-disproportionated state in the case of the thermally driven HS-LS transition in LaCoO_3_^41^.

We observed significant increase of the compressibility and hence decrease of bulk modulus from *K*_0_ ~ 200 (LP) to ~ 154 GPa (HP) for the fixed bulk modulus pressure derivative *K’* = 4. These results reveal an unusual softening of the lattice that follows the spin transition above ~ 50 GPa, which is in contrast to the anticipated hardening of the lattice at the HS-LS and/or Mott transition^6,11,12,20,24,25^. This also may be considered as a possible indirect confirmation of the idea of appreciable dynamical lattice effects. Furthermore, this agrees with the behavior of the octahedral FeO_6_ volume obtained above 50 GPa, which points to an averaged spin-state not consistent with the pure HS or LS one. While there is a possibility that quantum spin fluctuations are occurring on a time scale faster than our experimental measurements, we note that the pure HS and LS states are distinguished on the MS time-scales (~ 10^−7^ s) with no significant broadening observed.

Above ~ 108 GPa SC FeBO_3_ undergoes a distortion of the rhombohedral unit-cell resulting in a structural transition to the monoclinic *C2/c* phase, a subgroup of the original $R\overline{3}\mathrm{c }$. The phase transition is accompanied by an additional reduction of the Fe–O distances by ~ 1.3% and octahedral volume by ~ 3%. This suggests an additional electron density deformation and consequently redistribution of *3d* electrons on the Fe^3+^ site. Since the total octahedral volume drop accumulated throughout the different phase transitions up to ~ 108 GPa in FeBO_3_ is about 12.2% (i.e., in agreement with Shannon^36,39^), we can deduce that the $R\overline{3}\mathrm{c }$ to *C2/c* phase transition is associated with a completion of the HS/LS spin crossover for all Fe^3+^ in FeBO_3_. The *C2/c* phase is characterized by a reduced compressibility, as that expected for a complete LS state (Fig. 2a). Considering that the phase transformation at ~ 50 GPa is accompanied by a Mott insulator-to-semiconductor transition with a collapse of the charge gap from ~ 2.9 to 0.5 eV, we propose that the $R\overline{3}\mathrm{c }$ to *C2/c* phase transformation above ~ 106 GPa, associated with a completion of the Fe^3+^ transition into the LS state, may result in metallization of FeBO_3_.

In contrast to SC, in PWD FeBO_3_ characterized by strong deviatoric stress and grain-grain strain the structural transition is very sluggish, far from completion even at ~ 145 GPa. In agreement with this, PWD MS at ~ 140 GPa shows only some small decrease in the abundance of the HS state and of *H*_hf_. We believe that the above consideration is also applicable in the case of recent electrical transport measurements^19^ performed without any pressure-transmitting medium.

## Summary

We have shown that the interplay between electronic correlations, lattice, and spin states results in the formation of a complex electronic and magnetic behavior of FeBO_3_ under pressure. In particular, we observe a remarkable coexistence of the HS and LS states in the original $R\overline{3}\mathrm{c }$ structure, characterized by a single Fe^3+^ site, stable within a broad pressure range of ~ 50–106 GPa. We propose that the spin-disproportionated phase is driven by a cooperative ordering of the HS/LS states, randomly distributed over the $R\overline{3}\mathrm{c }$ lattice, suggesting a potential dynamical nature of the HS-LS correlations. This results in frustration of magnetic moments which is manifested by a large suppression of the Néel temperature to ~ 60 K above ~ 50 GPa, compare to *T*_N_ ~ 600 K at ~ 50 GPa before the transition^21^. Only above ~ 106 GPa for SC FeBO_3_ we found the transition to the lower symmetry *C2/c* phase which is associated with a further spin-state alteration and possible metallization of FeBO_3_. Our observations emphasize a remarkable importance of spin fluctuations and correlation effects for understanding the electronic structure and magnetic behavior of strongly correlated systems preceding the Mott transition. Overall, our results significantly improve understanding of the pressure-induced evolution of the electronic and magnetic properties of the Mott insulators. Our proposed novel scenario of the spin-state transformation may have important implications not only for the theoretical picture of compounds undergoing a spin-state transition, but also for understanding of quantum criticality of the Mott transitions. We believe that this topic deserves further detailed theoretical and experimental considerations.

## Methods

The experiments were performed with high-quality single crystals of FeBO_3_ (enriched to 96% with ^57^Fe isotope when needed for MS experiments)^43^. Polycrystalline samples were obtained by grinding the FeBO_3_ single crystal. Custom diamond anvil cells (DACs) and DACs of symmetric design were used to induce high pressure, with Ne, He or N_2_ serving as a pressure-transmitting medium. Pressure was determined using the ruby *R*_*1*_ fluorescence line as a pressure marker^44^, as well as the Ne, Au or Pt unit-cell volume in the case of various x-ray diffraction studies. ^57^Fe Mössbauer studies were performed using a 10 mCi ^57^Co (Rh) point source in a variable temperature (5–300 K) cryostat. The spectra at high pressures, 115 and 140 GPa, were collected using energy-domain synchrotron Mössbauer spectroscopy (SMS) carried out at the beamline ID18 at ESRF (Grenoble). SC XRD experiments were performed at the Extreme Conditions Beamline P02.2 at PETRA III (Hamburg, Germany), ID15B beamlines of ESRF (Grenoble) and the 13ID-D GSECARS beamline of APS (Argonne); PWD experiments at the ID27 and ID09A beamlines of ESRF and the 12.2.2 beamline of ALS (Berkeley). Further technical details about the methods used can be found in the Supplementary information S1.

## Supplementary Information

Supplementary Information.

## Data Availability

The data that support the findings of this study are available from the corresponding author upon reasonable request.
