# Supplementary material for: Pressure-induced high-spin/low-spin disproportionated state in the Mott insulator FeBO3
Source: Sci Rep. 2022 Jun 10;12:9647. doi: 10.1038/s41598-022-13507-4 (PMC9187741; doi:10.1038/s41598-022-13507-4)
Supplement: Supplementary file 1 — Supplementary Information. [file 41598_2022_13507_MOESM1_ESM.pdf]

Pressure-induced high-spin/low-spin disproportionated state in the Mott insulator $FeBO_3$

Weiming Xu,[1] Weiwei Dong,[2] Samar Layek,[1,3] Mark Shulman,[1] Konstantin Glazyrin,[2] Elena Bykova,[4] Maxim Bykov,[4] Volodymyr Svitlyk,[5] Michael Hanfland,[5] Moshe P. Pasternak,[1] Ivan Leonov,[6,7] Eran Greenberg,[1,8] Gregory Kh. Rozenberg[1]

[1]*School of Physics and Astronomy, Tel-Aviv University, 69978, Tel-Aviv, Israel*
[2]*Deutsches Elektronen Synchrotron (DESY), Notkestr. 85, 22607, Hamburg, Germany*
[3]*Department of Physics, School of Engineering, University of Petroleum and Energy Studies (UPES), Dehradun, Uttarakhand 248007, India*
[4]*Earth and Planets Laboratory, Carnegie Institution for Science, Washington, DC 20015, USA*
[5]*European Synchrotron Radiation Facility, BP220 38043 Grenoble, France*
[6]*M.N. Miheev Institute of Metal Physics, Russian Academy of Sciences, 620108 Yekaterinburg, Russia*
[7]*Ural Federal University, 620002 Yekaterinburg, Russia*
[8] *Applied Physics Division, Soreq NRC, Yavne, 81800, Israel*

Supplementary information

Experimental

Pressure was generated using TAU piston-cylinder DACs,[1] BX90-type DAC[2] or membrane cells with anvils having 120, 180 and 250 μm diameter culet size. Samples were loaded into a cavity of respectively ~60, 90 or 120 μm in diameter and about 15-30 μm thickness drilled in rhenium gaskets. In addition we used symmetric diamond anvil cells provided by PETRA-III (DESY, Germany), 150/300 μm 8° beveled Boehler-Almax diamonds. Neon, helium or nitrogen was used as a pressure medium, depending on an experiment. Along with the samples, small ruby balls were added for pressure measurement using the ruby fluorescence spectroscopy, the calibration scales mentioned in Ref. [3] were used for pressure determination. For XRD measurements Pt strips or Au powder were used for manometry as well as.

(a) Mössbauer Spectroscopy

^{57}Fe Mössbauer in-house measurements for polycrystalline sample were performed up to 85 GPa using a 10 mCi ^{57}Co (Rh) point source in a variable temperature (5 – 300 K) cryostat. The typical collection time for each spectrum was ~24 hours. Spectra were analyzed using appropriate fitting programs, e.g. [4], from which the hyperfine interaction parameters (internal magnetic field H_{hf}, isomer (centroid) shift IS and quadrupole splitting/shift QS) and the corresponding relative abundances of the spectral sub-components were derived. The isomer shift is calibrated relative to α-Fe at room-temperature (RT). The pressure uncertainties are ~5% of the reported average

pressure. The spectra at higher pressures, 115 and 140 GPa, were collected using energy-domain synchrotron Mössbauer spectroscopy (SMS) carried out at the beamline ID18 at ESRF at temperatures down to 3 K (see [5] for more details). These spectra were collected with the source at RT and, therefore, are affected by the 2nd order Doppler shift.

(b) X-ray diffraction

Powder XRD measurements were carried out at room temperature in angle-dispersive mode at pressures up to 55 GPa at the 12.2.2 beamline of ALS (Berkeley) and up to ~150 GPa at the ID27 beamline of ESRF (Grenoble) with a wavelength of λ = 0.4133 Å and 0.3738 Å, respectively. Diffraction images were collected using MAR345 image plates. The image data were integrated using the FIT2D and DIOPTAS programs[6,7,8] and the resulting diffraction patterns were analyzed by Rietveld refinement using GSAS[9] and EXPGUI[10] software.

Isobaric powder XRD low-temperature measurements were carried out at the ID09A beamline of ESRF (Grenoble) with a wavelength of λ = 0.415244 Å using MAR555 flat panel image plate and a *He* cryostat with cooling down to 10 K. *He* was used as a pressure medium and Pt as an in-situ XRD pressure marker. Pressure values were calculated taking into account Pt thermal expansion.[11]

Single crystal XRD

Sample preparation

Transparent light-green single plate-shaped crystals of $FeBO_3$ (the plane of the plate parallel the basal (111) plane) with an average size of 0.03 x 0.02 x 0.01 mm^3 were pre-selected on a three-circle Bruker diffractometer equipped with a SMART APEX CCD detector and a high-brilliance Rigaku rotating anode (Rotor Flex FR-D, Mo-*K*α radiation) with Osmic focusing X-ray optics.

Data collection

The single-crystal XRD experiments were conducted in four runs.

Run#1 was carried out at the 13-IDD beamline at the Advanced Photon Source (APS), Chicago, USA (MAR165 CCD detector, λ = 0.3344 Å, beam size 2(V) x 4(H) μm^2). Sample-to-detector distance, coordinates of the beam center, tilt angle and tilt plane rotation angle of the detector images were calibrated using LaB_6 powder. XRD wide images were collected during continuous rotation of DACs typically from –35 to +35 on omega; while XRD single-crystal data collection experiments were performed by narrow 0.5° scanning of the same omega range. DIOPTAS software[7] was used for preliminary analysis of the 2D images and calculation of pressure values from the positions of the XRD lines of Ne.

A single crystal of $FeBO_3$ with an average size of 0.03 x 0.02 x 0.01^3 mm together with a small ruby chip (for pressure estimation) were loaded into BX90-type DAC equipped with Boehler-Almax diamonds with 250 μm culet size. A hole with diameter about 120 μm in rhenium gasket pre-indented to 30 μm was served as a pressure chamber. Neon was used both as a pressure transmitting medium and as a pressure standard. Neon was loaded with a gas loading system installed at the Bayerisches Geoinstitut.[12]

The pressure was increased manually by tightening the screws. The DAC was compressed to 62.5(5) GPa with step 2-11 GPa with wide images being collected at each pressure point. The single-crystal XRD datasets have been collected only at 5 selected pressure points (namely at ambient pressure, 11.5(1), 45.8(3), 55.7(5) and 61.0(5) GPa) in order to determine orientation matrix of the single-crystal and refine the matrix after the phase transition. The indexing of the unit cell was performed using CrysAlisPro software[13]. Intensities of the diffraction reflections were extracted from the wide images by means of GSE_ADA/RSV package according to procedure described by P. Dera *et al.* [14]

Run#2 and #3 were carried out at the ID15B beamline at the European Synchrotron Radiation Facility (ESRF), Grenoble, France (MAR555 flat panel detector, λ = 0.41114 Å, beam size 10(V)x10(H) μm^2). Sample-to-detector distance, coordinates of the beam center, tilt angle and tilt plane rotation angle of the detector images were calibrated using Si powder. XRD wide images were collected during continuous rotation of DACs from –20 to +20 on omega; while data collection experiments were performed by narrow 0.5° scanning from –30 to +30 on omega. In **Run#2** the single-crystal of $FeBO_3$ was loaded in a membrane-driven DAC, with 300 μm Boehler-Almax diamond anvils. A hole with diameter about 150 μm in a steel gasket pre-indented to 35 μm was used as a pressure chamber. In **Run#3** the single-crystal of $FeBO_3$ was loaded in BX90-type DAC, with 250 μm Boehler-Almax diamond anvils. A hole with diameter about 120 μm in rhenium gasket pre-indented to 30 μm was used as a pressure chamber. Neon was used both as a pressure transmitting medium and as a pressure standard.

In **Run#2** the DAC was compressed to 36.1(3) GPa and in **Run#3** to 59.5(5) GPa.

Processing of XRD data (the unit cell determination and integration of the reflection intensities) were performed using CrysAlisPro software[13]. Empirical absorption correction was applied using spherical harmonics, implemented in the SCALE3 ABSPACK scaling algorithm, which is included in the CrysAlisPro software. A single crystal of an orthoenstatite $((Mg_{1.93},Fe_{0.06})(Si_{1.93},Al_{0.06})O_6$, *Pbca*, a = 18.2391(3), b = 8.8117(2), c = 5.18320(10) Å), was used to calibrate instrument model of CrysAlisPro software (sample-to-detector distance, the detector's

origin, offsets of the goniometer angles and rotation of the X-ray beam and the detector around the instrument axis).

Run#4 was carried out at the Extreme Conditions Beamline P02.2 at PETRA III (DESY, Germany) with a wavelength of 0.2898 Å with x-ray beam focused to 3(V) x 8(H) μm^2 (e.g. fullwidth at half maximum). Compression was conducted using the symmetric cells. Data was collected using Perkin Elmer XRD1621 flat panel detector. Preliminary calibration was done with CeO_2 and further refined using CrysAlisPro software employing a piece of the orthoenstatite coming from the same batch as in **Run#3.** Similar is the situation with absorption correction.

For measurements at P02.2, for the study exceeding 1 Mbar we used crystal smaller than 0.020 x 0.020 x 0.005 mm^3. We used beveled Boehler-Almax diamonds of 150/300 μm culet size. In contrast to the other runs, in order to boost the signal to noise ratio we used an amorphous metal insert (with an initial hole of 75 μm) into a Re gasket. Such geometry of a sample chamber produces lower scattering even if the gasket is hit by the tails of a larger x-ray beam. Prior to compression, Ne was loaded as pressure medium.

The following sequence was recorded during the spin state transition.

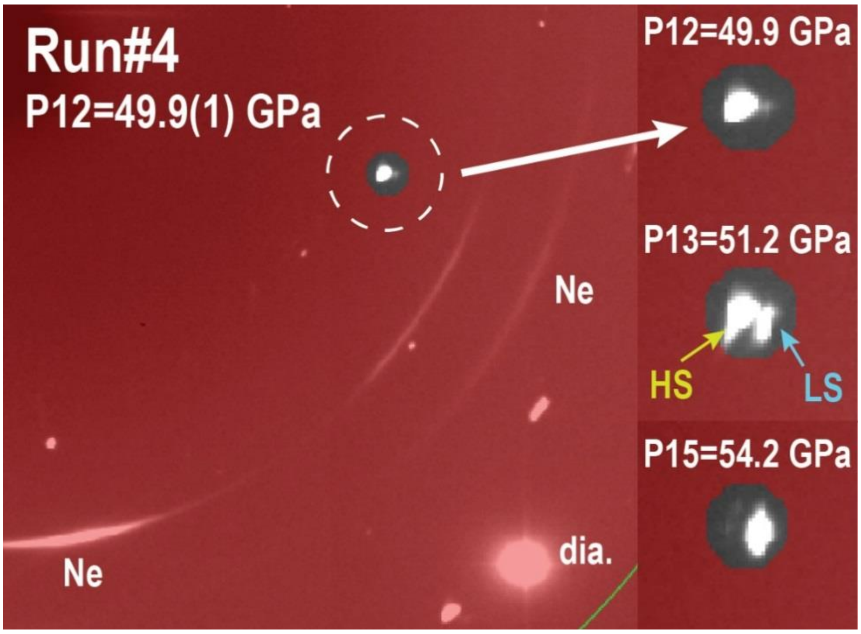

Fig. S1. 2D diffraction patterns (collected with DAC oscillation of ±20º) indicating diffraction response from $FeBO_3$ with a focus given to a specific diffraction spot changing as a function of pressure. HS and LS contributions are present at lower 2θ and higher 2θ, respectively. Center of the diffraction pattern is in the direction of the top left corner of the diffractogram. At ~50 GPa we observed a negligible contribution from LS HP state. At ~51.2(1) GPa the abundance from the low spin state has increased. HS LP almost disappeared at ~54.2(1) GPa as most of the intensity is transferred into the LS state. Certain diamond and Ne diffraction lines are indicated.

Structure solution and refinement of $FeBO_3$

At ambient pressure $FeBO_3$ adopts crystal structure of calcite ($CaCO_3$, sp.gr. $R\bar{3}c$, Z = 6) and all atoms are located on special positions: Fe atom occupies Wycoff position 6*b* (0, 0, 0), B - 6*a* (0, 0, 0.25) and oxygen is located on 18*e* (*x*, 0, 0.25). Therefore, only one oxygen coordinate, thermal parameters and scale factor have to be refined. Since body of the diamond anvil cell shadows more than 50% of the diffraction reflections, the reflection datasets are incomplete. In order to improve data/parameter ratio, we refined atomic thermal parameters in isotropic approximation. The structures were refined by full-matrix least squares against F^2 using the SHELXL-2014/7[15] (at certain points using OLEX2[16] frontend) and JANA2006[17] software.

The detailed summary of the crystal structure refinements along with information on unit cell parameters, atomic coordinates and isotropic displacement parameters are summarized in Tables 1, 2 and 3. Polyhedral volumes were determined with VESTA software[18]. Our SC-XRD data can be considered as a new reference for the $FeBO_3$ phase diagram and for the *V(P)* data.

SC versus PWD XRD data

Comparison of our SC and PWD data, and PWD data published in literature (e.g. [19]) indicate an appreciable difference in their *V(P)* behavior. This discrepancy may be related to differences in the initial state of sample material on the microscale. Following the recent discussion[20], considering PWD case one may expect additional strains or stresses due to grain-grain interactions. Particularly, some strongly correlated systems demonstrating spin state transitions are exceptionally sensitive to the effects of deviatoric stress and strain effects on the grain-grain interactions occurring in sample chambers of DACs.[20] These effects may induce a distortion of the structure, adjustment of local crystal field at Fe^{3+} sites, presumably affecting the onset of spin crossover. In addition, the grinding process, which is essential for powder preparation, should significantly increase the defect concentration in the grains and, thus, induce additional microstrain fields in the vicinity of Fe ions. In contrast, in a SC study one minimizes the contribution from undesirable effects of grain boundaries, intergrain stresses, and grinding-induced defects, and thus transform the materials to a configuration closer to thermodynamic equilibrium[20] (for a more detailed discussion of this problem see also [21]).

We note that in the HS LP phase the increasing deviation of the PWD *V(P)* data from SC EOS (Fig. 2a) suggests a growing impact of strains and stresses with pressure increase. Meanwhile, the spin transition around 50 GPa results in converging of the relative volume values observed in SC and PWD experiments. The comparison of the SC EOS and PWD data at the range ~60 – 115 GPa show that the volume difference ΔV remains stable (see Fig. S5) suggesting that the contribution

from undesirable stresses and strains does not change appreciably with pressure increase up to ~110 GPa. However, at ~110 GPa one observes a drastic increase of discrepancy of the SC and PWD data: while SC shows the sharp transition to the *C2/c* structure, PWD data demonstrate a very sluggish structural transformation, whose onset is indicated by a sharp increase of the ΔV value above 110 GPa (Fig. S5). Such a change of the *V(P)* behavior suggests an onset of a structural transformation related to a significantly increased strain resulting in growing discrepancies with a hydrostatic reference, e.g. SC data.

Figures and Tables

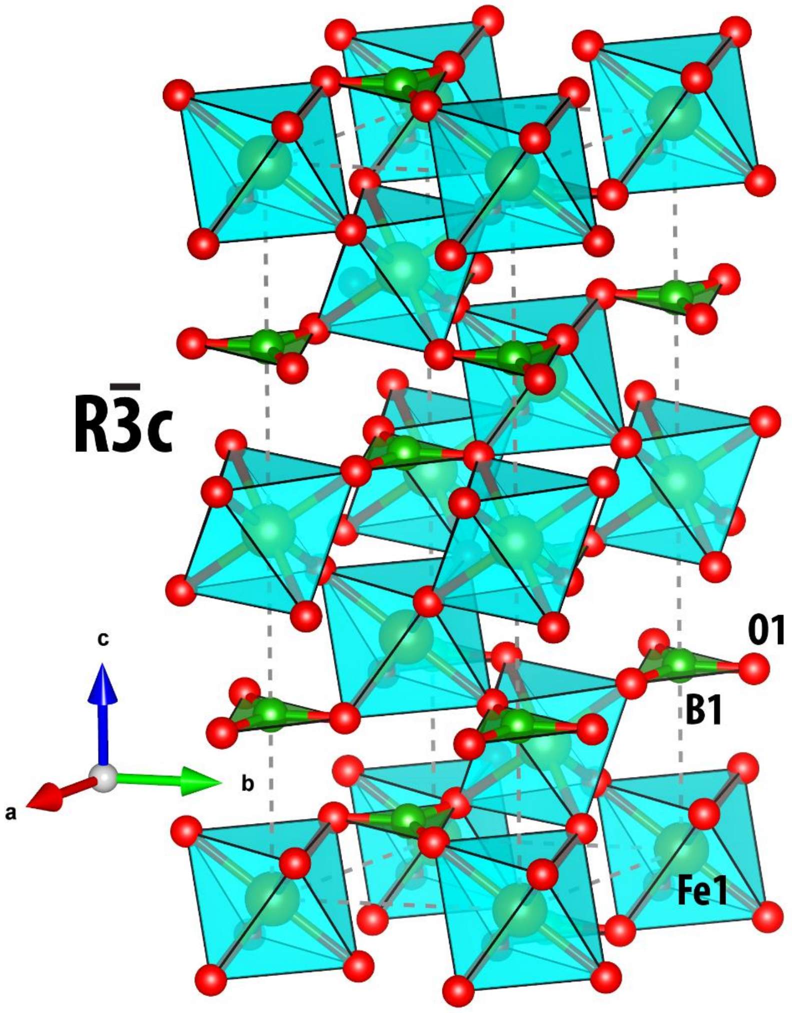

Fig. S2. The low-pressure crystal structure of $FeBO_3$ with the $R\bar{3}$c symmetry. The red and green spheres correspond to the O, and B atoms. Image was prepared using VESTA software package.[18]

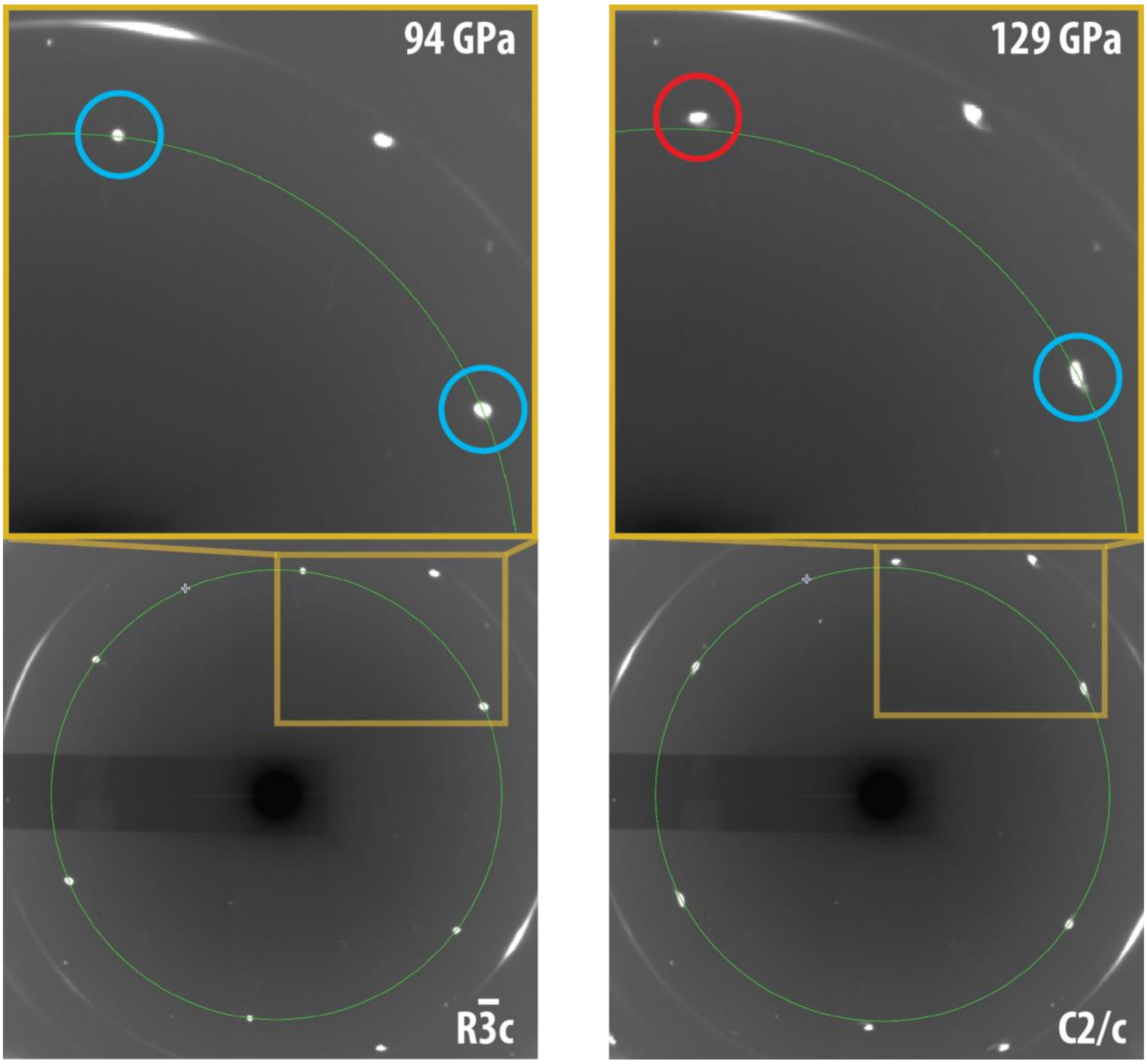

Fig. S3. X-ray single crystal diffraction patterns of $FeBO_3$ at RT at various pressures (λ = 0.2898 Å). Note a splitting of the $(10\bar{2})$ reflection in the XRD pattern at 129 GPa, signifying lowering of the original symmetry. Picture was prepared with the help of DIOPTAS.[8]

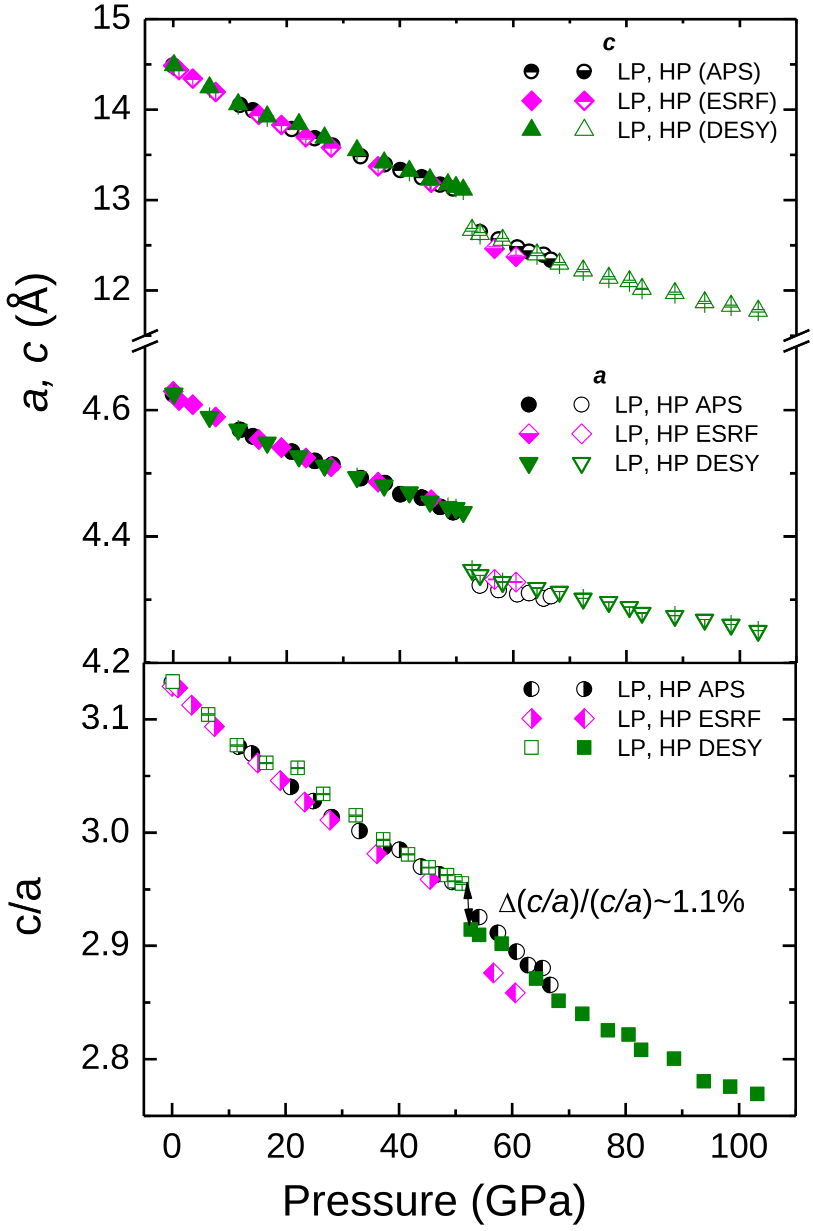

Fig. S4. Pressure dependence of the lattice parameters *a*, *c* and *c/a* ratio for $R\bar{3}c$ phase of $FeBO_3$.

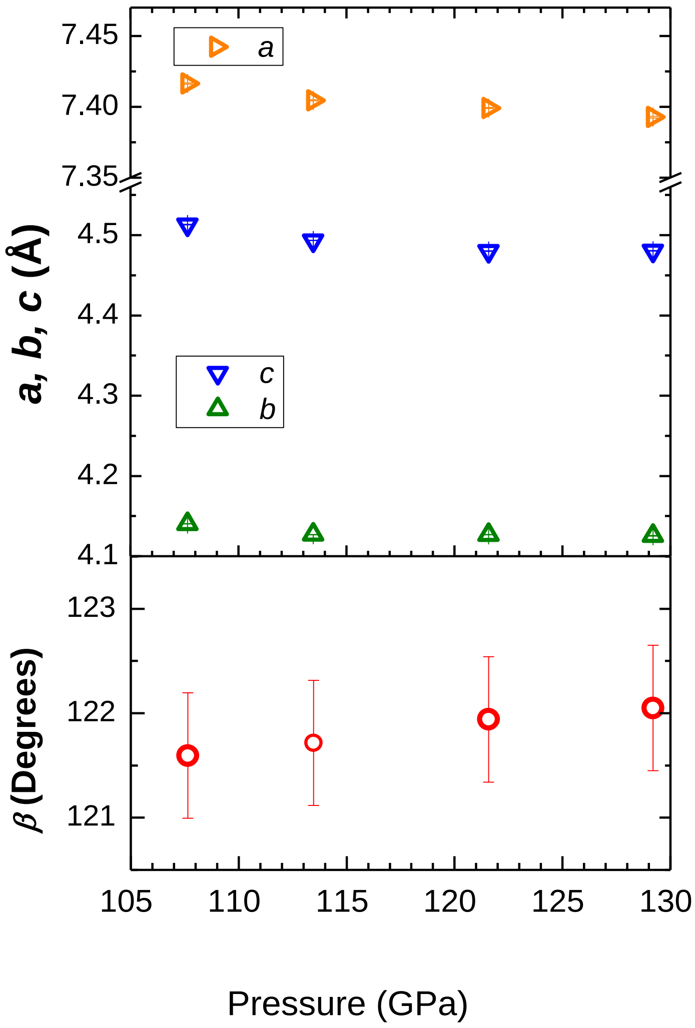

Fig. S5. Pressure dependence of the lattice parameters *a*, *b*, *c* and *β* angle for *C2/c* phase of $FeBO_3$.

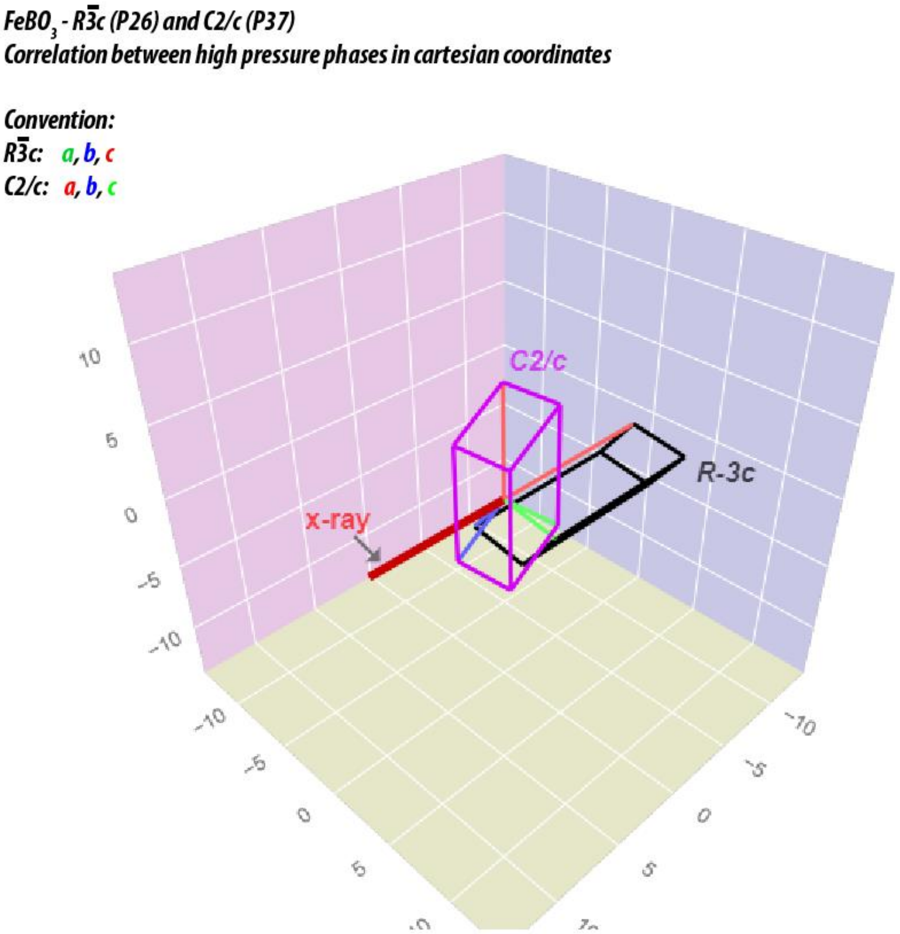

Fig. S6. Crystallographic unit cell relation between $R\bar{3}$c (black frame) and *C2/c* (purple frame) in cartesian coordinates, direct space. P26 and P37 correspond to 103.1(1) and 129.2(1) GPa, respectively. Representation units correspond to Å. Upon loading into sample chamber, the orientation of the crystal was such that the *c* axis of $R\bar{3}$c was directed along compression axis as indicated by x-ray beam direction. After the transformation to *C2/c*, *a* axis of *C2/c* was directed perpendicular to *c* of $R\bar{3}$c and *c* axis of *C2/c* was oriented in the direction very similar to *a* axis of $R\bar{3}$c. Picture was prepared using python3 and plotly library using information of crystal orientation determined with CrysAlisPro.[13] The code is derived from the open source K. Glazyrin's github repository located at https://github.com/lorcat/Crysalis-CIFOD.

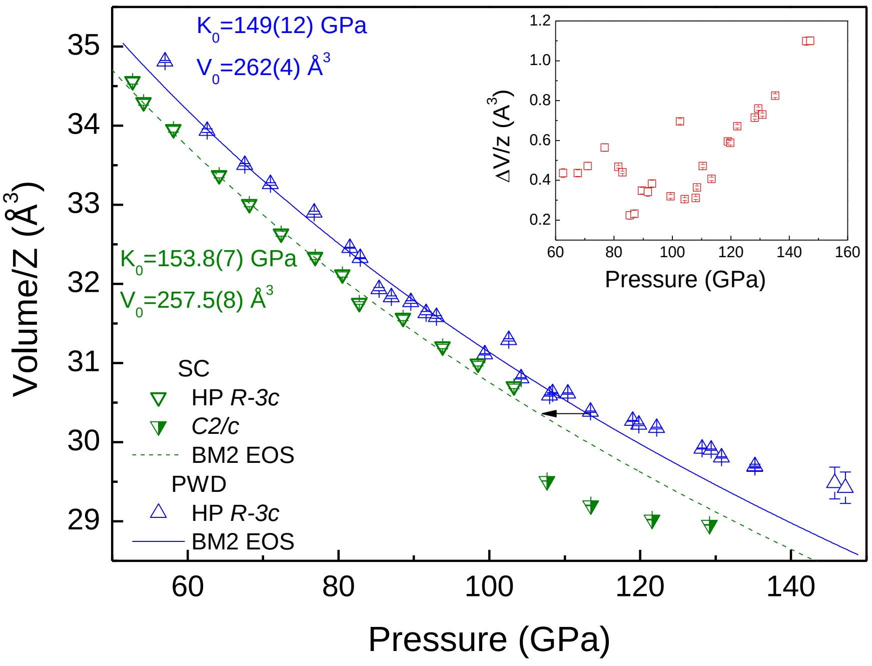

Fig. S7. Pressure dependencies of the unit-cell volume divided by *Z* unit formulas (Z=6 and 4 for the $R\bar{3}c$ and *C2/c* phases, respectively), determined in the powder and single crystal XRD studies at the pressure range 55 – 150 GPa. The inset shows the difference in the volume values $\Delta V/z$ obtained in the PWD and SC experiments considering HP $R\bar{3}c$ phase EOS as a reference line.

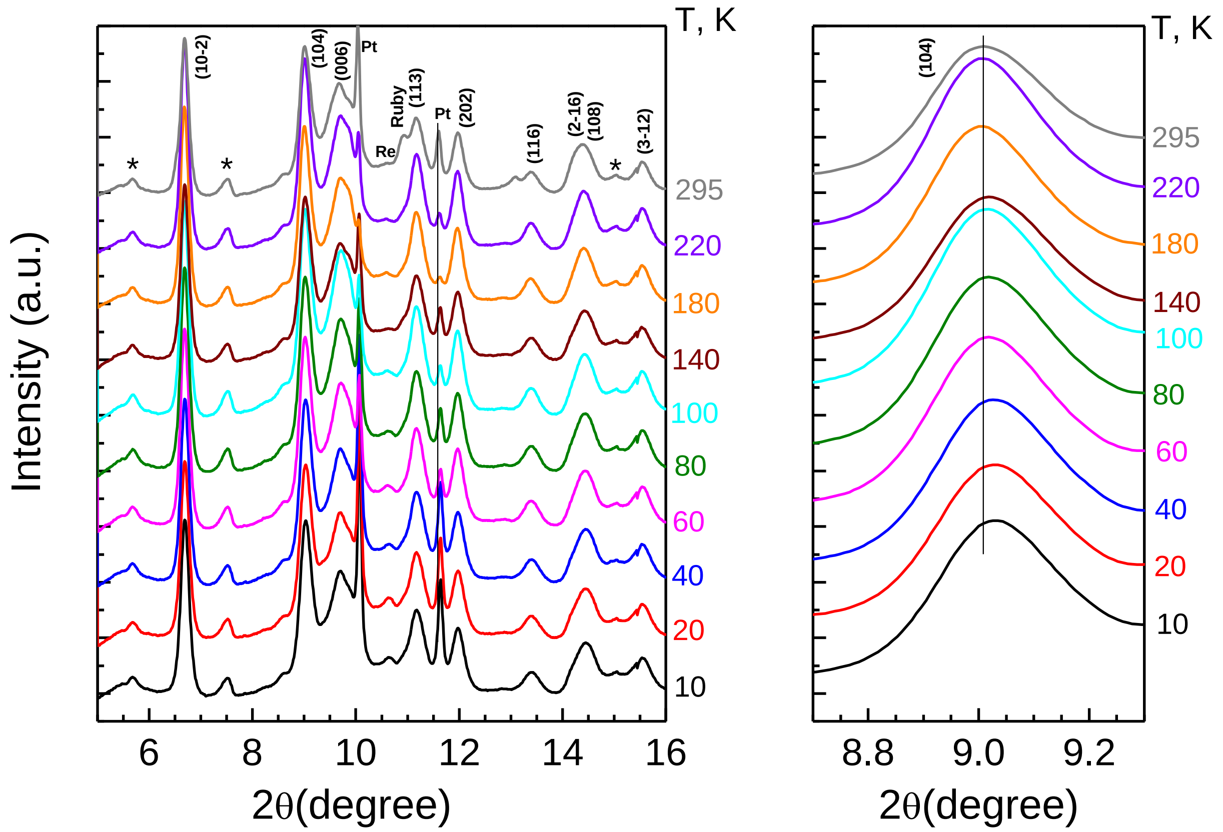

Fig. S8. X-ray powder diffraction patterns of $FeBO_3$ at 78 GPa at various temperatures (λ = 0.3738 Å). Note a non-monotonous shift of $FeBO_3$ peaks with temperature decrease in contrast to an expected monotonous shift right of Pt peak around 11.6°. A part of the spectrum in the 2θ range of 8.7°–9.3° is expanded in the right panel to emphasize this unusual behavior of the (104) reflection. The diffraction peaks of the cryostat window (not changing their position with temperature variation) are marked with an asterisk.

Table 1. Details of typical single crystal structure refinements for $FeBO_3$ at high pressures (Runs#1)* as provided by SHELXL

**Pressure, GPa**	**0.0001^{LP}**	**24.2(4)^{LP}**	**46.8(5)^{LP}**	**64.8(5)^{HP}**
***a* (Å)**	4.6248(4)	4.5189(6)	4.4436(12)	4.3055(17)
***c* (Å)**	14.4862(12)	13.6808(19)	13.167(3)	12.337(5)
***V* (Å^3)**	268.33(5)	241.94(7)	225.16(13)	198.06(17)
**Reflections collected**	320	264	164	155
**Independent reflections**	82	60	56	54
**Independent reflections [$I > 2\sigma(I)$]**	81	60	56	53
**Refined parameters**	5	5	5	5
$R_{int}(F^2)$	0.1522	0.0853	0.1194	0.1186
$R(\sigma)$	0.0656	0.0493	0.082	0.0709
R_1 [$I > 2\sigma(I)$]	0.0873	0.0387	0.0464	0.035
wR_2 [$I > 2\sigma(I)$]	0.1774	0.0739	0.0949	0.0819
R_1	0.0888	0.0387	0.0464	0.0385
wR_2	0.1788	0.0739	0.0949	0.0823
**Goodness of fit on F^2**	1.214	1.204	1.26	1.288
$\Delta\rho_{max}$(e / Å^3)	1.68	0.987	1.093	1.195
$\Delta\rho_{min}$(e / Å^3)	-1.89	-1.532	-1.252	-1.473
x(O)	0.296(2)	0.3008(11)	0.3025(8)	0.3108(9)
U_{iso}(Fe) (Å^2)	0.0034(6)	0.0044(4)	0.0041(4)	0.0049(4)
U_{iso}(B) (Å^2)	0.007(3)	0.0046(16)	0.0038(15)	0.0066(15)
U_{iso}(O) (Å^2)	0.0064(15)	0.0060(9)	0.0052(8)	0.0062(7)
d(Fe−O) (Å)	2.031(4)	1.952(2)	1.9025(18)	1.8070(19)
d(B−O) (Å)	1.371(10)	1.359(5)	1.344(4)	1.338(4)

*$FeBO_3$ adopts calcite crystal structure, space group $R\bar{3}c$, $Z = 6$, atoms' Wyckoff positions:

Fe 6*b* (0, 0, 0)

B 6*a* (0, 0, 0.25)

O 18*e* (*x*, 0, 0.25)

Data collection: APS, 13-IDD beamline, MAR165 CCD detector, λ = 0.3344 Å

Table 2. Details of typical single crystal structure refinements for $FeBO_3$ at high pressures (Runs#2, 3)* as provided by SHELXL

**Pressure, GPa**	**0.0001**[LP]	**23.4(3)**[LP]	**45.5(5)**[LP]	**59.5(5)**[HP]
a **(Å)**	4.62920(10)	4.5242(2)	4.4581(7)	4.3275(6)
c **(Å)**	14.4849(7)	13.694(11)	13.19(3)	12.37(2)
**V (Å^3)**	268.818(17)	242.75(19)	227.1(4)	200.6(3)
**Reflections collected**	80	144	162	127
**Independent reflections**	54	46	45	37
**Independent reflections $[I > 2\sigma(I)]$**	51	46	42	37
**Refined parameters**	5	5	5	5
**$R_{int}(F^2)$**	0.0619	0.036	0.0602	0.0619
**$R(\sigma)$**	0.0535	0.025	0.0344	0.0323
**R_1 $[I > 2\sigma(I)]$**	0.0638	0.047	0.0553	0.0598
**wR_2 $[I > 2\sigma(I)]$**	0.146	0.1168	0.1392	0.1378
**R_1**	0.0644	0.047	0.0559	0.0598
**wR_2**	0.1482	0.1168	0.1437	0.1378
**Goodness of fit on F^2**	1.215	1.228	1.254	1.159
**$\Delta\rho_{max}(e$ / Å$^3)$**	1.111	0.902	1.543	1.102
**$\Delta\rho_{min}(e$ / Å$^3)$**	-1.504	-0.606	-1.061	-1.038
**x(O)**	0.2986(8)	0.3011(6)	0.3027(9)	0.3108(8)
**U_{iso}(Fe) (Å^2)**	0.0036(11)	0.0042(7)	0.0064(9)	0.0069(10)
**U_{iso}(B) (Å^2)**	0.009(4)	0.005(2)	0.008(3)	0.014(5)
**U_{iso}(O) (Å^2)**	0.0047(12)	0.0042(8)	0.0087(12)	0.0096(12)
**d(Fe−O) (Å)**	2.0278(18)	1.9539(14)	1.907(3)	1.8148(19)
**d(B−O) (Å)**	1.382(4)	1.362(3)	1.349(5)	1.345(4)
**CCDC Deposition #**	2109033	2109041	2109042	2109044

*$FeBO_3$ adopts calcite crystal structure, space group $R\bar{3}c$, Z = 6, atoms' Wyckoff positions:

Fe 6*b* (0, 0, 0)
B 6*a* (0, 0, 0.25)
O 18*e* (*x*, 0, 0.25)

Data collection: ESRF, ID15B beamline, MAR555 flat panel detector, λ = 0.41114 Å
The complete list of CCDC deposition numbers for runs #2, 3 is: 2109033- 2109044

Table 3. Details of typical single crystal structure refinements for $FeBO_3$ at high pressures (Run#4)* as provided by JANA2006

Pressure, GPa	11.5(1)^{LP}	32.4(4)^{LP}	49.9(1)^{LP}	58.1(1)^{HP}	103.3(1)^{HP}
a (Å)	4.5690(2)	4.4940(2)	4.4450(2)	4.3281(2)	4.2510(2)
c (Å)	14.058(7)	13.550(6)	13.144(6)	12.559(14)	11.772(12)
V (Å^3)	254.16(13)	236.98(11)	224.90(10)	203.7(2)	184.2(2)
Reflections collected	265	287	281	231	207
Independent reflections [$I > 2\sigma(I)$]	64	61	58	41	42
Refined parameters	5	5	5	5	5
R_1 [$I > 2\sigma(I)$]	0.0261	0.0400	0.0425	0.0457	0.0566
wR_2 [$I > 2\sigma(I)$]	0.0669	0.0841	0.0859	0.0897	0.1190
$R_{int}(F^2)$	0.0242	0.0394	0.0491	0.0593	0.0424
x(O)	0.2997(5)	0.3011(6)	0.3019(6)	0.3102(9)	0.3093(10)
U_{iso}(Fe) (Å^2)	0.0033(3)	0.0037(3)	0.0039(4)	0.0006(5)	0.0037(6)
U_{iso}(B) (Å^2)	0.0032(11)	0.0053(13)	0.0061(15)	0.0017(19)	0.010(3)
U_{iso}(O) (Å^2)	0.0044(5)	0.0045(5)	0.0048(6)	0.0006(8)	0.0051(9)
d(Fe−O) (Å)	1.988(1)	1.938(1)	1.903(2)	1.825(2)	1.768(2)
d(B−O) (Å)	1.369(2)	1.353(3)	1.342(3)	1.343(4)	1.315(4)
CCDC Deposition #	2114258	2114259	2114260	2114261	2114262

*$FeBO_3$ adopts calcite crystal structure, space group $R\bar{3}c$, $Z = 6$, atoms' Wyckoff positions:

Fe 6*b* (0, 0, 0)

B 6*a* (0, 0, 0.25)

O 18*e* (*x*, 0, 0.25)

Data collection: DESY, P02.2 beamline, XRD1621 flat panel detector, $\lambda = 0.2898$ Å

Table 3. Details of typical single crystal structure refinements for $FeBO_3$ at high pressures (Run#4)* as provided by JANA2006

**Pressure, GPa**	**129.2(1)^{HP2}**
a **(Å)**	7.3926(5)
b **(Å)**	4.1253(4)
c **(Å)**	4.4806(3)
β (Degrees)	122.0(6)
V **($Å^3$)**	115.8(8)
**Reflections collected**	252
**Independent reflections [$I > 2\sigma(I)$]**	80
**Refined parameters**	10
**R_1 [$I > 2\sigma(I)$]**	0.0540
**wR_2 [$I > 2\sigma(I)$]**	0.1179
**$R_{int}(F^2)$**	0.0282
**y(B)**	0.2522(15)
y**(O1)**	0.5681(11)
x**(O2)**	0.1533(11)
y**(O2)**	0.0960(9)
z**(O2)**	0.248(3)
**U_{iso}(Fe) ($Å^2$)**	0.0089(5)
**U_{iso}(B) ($Å^2$)**	0.012(2)
**U_{iso}(O1) ($Å^2$)**	0.0093(11)
**U_{iso}(O2) ($Å^2$)**	0.0105(8)
d**(Fe−O) (Å)§**	1.732(9)
d**(B−O) (Å)§**	1.305(13)
**CCDC Deposition #**	2114263

§ - average bond distance

*$FeBO_3$ adopts space group *C2/c*, *Z* = 4, atoms' Wyckoff positions:

Fe 4c (0.25, 0.25, 0)
B 4e (0, y, 0.25)
O1 4e (0, y, 0.25)
O2 8f (*x*, y, z)

Data collection: DESY, P02.2 beamline, XRD1621 flat panel detector, λ = 0.2897 Å

References:

[1] G. Yu. Machavariani, M. P. Pasternak, G. R. Hearne, and G. Kh.Rozenberg, "A multipurpose miniature piston-cylinder diamond-anvil cell for pressures beyond 100 GPa", Rev. Sci. Instrum. **69**, 1423 (1998).

[2] I. Kantor *et al.*, "BX90: A new diamond anvil cell design for X-ray diffraction and optical measurements", Rev. Sci. Instrum. **83**, 125102 (2012).

[3] A. Dewaele, M. Torrent, P. Loubeyre and M. Mezouar, "Compression curves of transition metals in the Mbar range: Experiments and projector augmented-wave calculations", Phys. Rev. B **78**, 104102 (2008).

[4] C. Prescher, C. McCammon, and L. Dubrovinsky, "MossA: a program for analyzing energy-domain Mössbauer spectra from conventional and synchrotron sources", Journal of Applied Crystallography, **45**(2), 329-331 (2012).

[5] V. Potapkin *et al.*, "The ^{57}Fe Synchrotron Mössbauer Source at the ESRF", J. Synchrotron Radiat. **19**, 559 (2012).

[6] A. P. Hammersley, "FIT2D: An Introduction and Overview", ESRF Internal Report, ESRF97HA02T (1997).

[7] A. P. Hammersley, S. O. Svensson, M. Hanfland, A. N. Fitch, and D. Hausermann, "Two-dimensional detector software: from real detector to idealised image or two-theta scan", High Pressure Research, **14**, 235 (1996).

[8] C Prescher and V. B. Prakapenka, "DIOPTAS: a program for reduction of two-dimensional X-ray diffraction data and data exploration", High Press. Res. **35,** 223–230 (2015).

[9] A. C. Larson, R. B. Von Dreele, "General Structure Analysis System (GSAS)", Los Alamos National Laboratory Report LAUR 86-748 (2000).

[10] B. H. Toby, "*EXPGUI*, a graphical user interface for *GSAS*", J. Appl. Crystallography **34**, 210 (2001).

[11] F. C. Nix, D. Macnair, "The Thermal Expansion of Pure Metals. II: Molybdenum, Palladium, Silver, Tantalum, Tungsten, Platinum, and Lead", Physical Review **61**, 74 (1942); G. K. White, "Thermal expansion of platinum at low temperatures", J. Phys. F; Metal Phys. **2**, L30 (1972).

[12] A. Kurnosov *et al.*, "A novel gas-loading system for mechanically closing of various types of diamond anvil cells", Rev. Sci. Instrum. **79**, 045110 (2008).

[13] CrysAlisPro Software system, version 1.171.37.35, Rigaku Oxford Diffraction, Oxford, UK. (2014).

[14] P. Dera *et al.*, "High pressure single-crystal micro X-ray diffraction analysis with GSE-ADA/RSV software", High Press. Res. **33**, 466–484 (2013).

[15] G. M. Sheldrick, "Crystal structure refinement with SHELXL", Acta Crystallogr. Sect. C Struct. Chem. **71**, 3–8 (2015).

[16] O. V. Dolomanov, L. J Bourhis, R. J. Gildea, J. A. Howard, and H. Puschmann, "OLEX2: a complete structure solution, refinement and analysis program", Journal of Applied Crystallography **42**, 339-341 (2009).

[17] V. Petříček, M. Dušek, and L. Palatinus, "Crystallographic computing system JANA2006: general features", Zeitschrift für Kristallographie-Crystalline Materials **229**, 345-352 (2014).

[18] K. Momma and F. Izumi, "VESTA 3 for three-dimensional visualization of crystal, volumetric and morphology data", J. Appl. Crystallogr. **44**, 1272–1276 (2011).

[19] A. G. Gavriliuk *et al.*, **“**Equation of State and Structural Phase Transition in $FeBO_3$ at High Pressure”, JETP Letters **75**, 23 (2002).

[20] K. Glazyrin, N. Miyajima, J. S. Smith, K. K. M. Lee, "Compression of a multiphase mantle assemblage: Effects of undesirable stress and stress annealing on the iron spin state crossover in ferropericlase", J. Geophys. Res.: Solid Earth **121**, 3377 (2016); K. Glazyrin *et al.*, “Critical behavior of $Mg_{1-x}Fe_xO$ at the pressure-induced iron spin-state crossover”, Phys. Rev. B **95**, 214412 (2017).

[21] K. Glazyrin et al., to be published.
